# Designing Cost-Sharing Methods for Bayesian Games

**DOI:** 10.1007/s00224-017-9832-3

**Published:** 2017-12-20

**Authors:** George Christodoulou, Stefano Leonardi, Alkmini Sgouritsa

**Affiliations:** 1 Computer Science Department, University of Liverpool, Liverpool, UK; 2 Department of Computer, Control and Management Engineering Antonio Ruberti, Sapienza University of Rome, Rome, Italy

**Keywords:** Price of anarchy, Bayesian games, Network design, Cost-sharing games

## Abstract

We study the design of cost-sharing protocols for two fundamental resource allocation problems, the *Set Cover* and the *Steiner Tree Problem*, under environments of incomplete information (Bayesian model). Our objective is to design protocols where the worst-case Bayesian Nash equilibria have low cost, i.e. *the Bayesian Price of Anarchy (PoA)* is minimized. Although budget balance is a very natural requirement, it puts considerable restrictions on the design space, resulting in high PoA. We propose an alternative, relaxed requirement called budget balance in the equilibrium (BBiE). We show an interesting connection between algorithms for *Oblivious Stochastic* optimization problems and cost-sharing design with low PoA. We exploit this connection for both problems and we enforce approximate solutions of the stochastic problem, as Bayesian Nash equilibria, with the same guarantees on the PoA. More interestingly, we show how to obtain the same bounds on the PoA, by using *anonymous* posted prices which are desirable because they are easy to implement and, as we show, induce *dominant strategies* for the players.

## Introduction

A *cost-sharing game* is an abstract setting that describes interactions of selfish players in environments where the cost of the produced solution needs to be shared among the participants. A *cost-sharing protocol* prescribes how the incurred cost is split among the users. This defines a game that is played by the participants, who try to select outcomes that incur low personal costs. Chen, Roughgarden and Valiant [23] initiated the *design* aspect, seeking for protocols that induce approximately efficient equilibria, *with low Price of Anarchy (PoA)* [55]. Similarly, we study the design of cost-sharing protocols, for two well-studied and very general resource allocation problems with numerous applications, the *Set Cover* and the *Steiner tree (multicast)* problem.

### Set Cover Game

In the (weighted) set cover problem, there is a universe of *n* elements, *U* = {1, …, *n*}, and a family of subsets of *U*, $\mathscr {F}=\{F_{1},\ldots , F_{m}\}$, with weights/costs $c_{F_{1}},\ldots , c_{F_{m}}$. A subset of elements, *X* ⊆ *U*, needs to be covered by the *Fi*′*s* so that the total cost is *minimized*. We are interested in a game theoretic version, where there are |*X*| players and |*U*| possible *types*; each player’s type associates her with a specific element of *U* and *X* corresponds to the set of players’ types. Multiple players may have the same type. A player’s action is to chose a subset from $\mathscr {F}$ that covers her element, and pay some cost-share for using it. A cost-sharing method prescribes how the subsets’ costs are split among players.

### Multicast Game

In a multicast game, there is a rooted (connected) undirected graph *G* = (*V*, *E*, *t*), where each edge *e* carries a nonnegative weight *c*_*e*_ and *t* is a designated root. There are *k* players and |*V* | = *n* possible types; each player’s type associates him with a specific vertex of *V* which needs to establish connectivity with *t*. The players’ strategies are all the paths that connect their terminal with *t*. A cost-sharing method defines the cost-shares of the players.

### Cost-Sharing under Uncertainty

There are two different possible sources of uncertainty that may need to be considered in the above scenarios. Firstly, the designer needs to specify the cost-sharing protocol, having only partial information about the players’ types. Moreover, the players themselves, when they select their actions, may have incomplete knowledge about the types of the other players. We approach the former by using a stochastic model similar to [29], and the latter, as a *Bayesian game*, introduced by [48], which is an elegant way of modelling selfishness in partial-information settings. In a Bayesian game, players do not know the private types of the other players, but only have *beliefs*, expressed by probability distributions over the possible realizations of the types.

The order of events is as follows; first, the designer specifies the cost-sharing methods, using the product probability distribution over the players’ types, then the players interact in the induced Bayesian game, and end up in a Bayesian Nash Equilibrium. We are interested in the design of protocols, where *all* equilibria have low cost i.e., the (Bayesian) PoA of the induced game is *low*.

### Budget Balance in the Equilibrium (BBiE)

One of the axioms that [23] required in their design space, that every cost-sharing protocol should satisfy, is budget balance i.e., that the players’ cost-shares cover exactly the cost of *any* solution. Although budget balance is a very natural requirement, it puts considerable restrictions on the design space. However, since we expect that the players will end up in a Nash equilibrium, it is not clear why one should be interested to impose budget balance in non-equilibrium states; the players are going to deviate from such states anyway. We propose an alternative, relaxed requirement that we call budget balance in the equilibrium (BBiE). A BBiE cost-sharing protocol satisfies budget balance in *all equilibria*; for any non-equilibrium profile we do not impose this requirement. This natural relaxation enlarges the design space but maintains the desired property of balancing the cost in the equilibrium. More importantly, this amplification of the design space, allows us to design protocols that dramatically outperform the best possible PoA bounds obtained by budget-balanced protocols. Indeed, by restricting to budget-balanced protocols, a lower bound of Ω(*n*) exists, for the complete information set cover game [23]; we extend this lower bound for the Bayesian setting. We further show a lower bound of ${\Omega }(\sqrt {n})$, for the multicast Bayesian game. We demonstrate that, by designing BBiE protocols, we can enforce better solutions, that dramatically improve the PoA. For the set cover game, we improve the PoA to *O*(*n*/log *n*) (or *O*(log *n*) if *m* = *poly*(*n*)). Regarding the multicast game, we improve the PoA to O(1).

### Posted Prices

It is a very common practice, especially in large markets and double auctions, for sellers to use posted prices. More closely to cost-sharing games is the model proposed by Kelly [51] regarding *bandwidth allocation*. Kelly’s mechanism processes players’ willingness to pay and posts a price for the whole bandwidth. Then each player pays a price proportional to the bandwidth she uses. This can be seen as pricing an infinitesimal quantity of bandwidth and the players, acting as price-takers, choose some number of quantities to buy. It turns out that it is in the best interest of the players to buy the whole bandwidth.

The use of posted prices, to serve as cost-sharing mechanism, is highly desirable, but not always possible to achieve; a price is posted for each resource and then the players behave as price takers, picking up the cheapest possible resources that satisfy their requirements. Such a mechanism is desirable because it is extremely easy to implement and also induces *dominant strategies*. We stress that our main results can be implemented by *anonymous* posted prices.

### Results and Discussion

We study the design of cost-sharing protocols for two fundamental resource allocation problems, the *Set Cover* and the *Steiner tree problem*. We are interested in environments of incomplete information where both the designer and the players have partial information, described by prior probability distributions over types. Our objective is to design cost-sharing protocols that are *BBiE* and the worst-case equilibria have low cost, i.e. *the Bayesian PoA* is minimized.

We show an interesting connection between algorithms for *ObliviousStocha-stic* optimization problems and cost-sharing design with low PoA. We exploit this for both problems and we are able to enforce approximate solutions of the stochastic problem, as Bayesian Nash equilibria, with the same guarantees on the PoA. Although this connection is quite simple, it results in significant improvement on the PoA comparing to budget-balanced protocols. More precisely, we map each player to a *single* specific strategy and charge very high costs for any alternative strategy. In this way, their mapped strategy becomes a (strongly) *dominant strategy*. For the set cover game, we enforce the oblivious solution given by [45]. They apriori map each player *i* to some subset $F_{i} \in \mathscr {F}$; then, if *i* is sampled, *F*_*i*_ should be in the induced solution. For the multicast game, the algorithm of [42], for the online Steiner tree problem, provides an oblivious solution.

#### Budget-Balanced Protocols

(Section 3). First, we provide lower bounds for the PoA of budget-balanced protocols. It is not hard to see that there exists a set cover game that reduces to the lower bound of Chen, Roughgarden and Valiant [23] for the multicast directed network games, resulting in PoA= Ω(*n*) in the complete information case; (see Appendix for the reduction). For the stochastic or Bayesian setting, where players are *i.i.d.*, we show that the same lower bound holds. Regarding the multicast game, the PoA is *O*(1) for the complete information case [23] and the stochastic case [29, 42]. However, we show that for the Bayesian setting there is a lower bound of ${\Omega }(\sqrt {n})$ (see Table 1 for a summary).

**Table 1:** PoA of budget-balanced protocols

	Budget-balanced protocols			
	Set cover		Multicast	
Complete information	Θ(*n*)	[23]	*O*(1)	[23]
Bayesian	Ω(*n*)		${\Omega }(\sqrt {n})$	

#### BBiE Protocols

(Section 4). For the Bayesian (and stochastic) set cover game there exists an *ex-post*^1^ BBiE protocol (determined in polynomial time) with PoA of *O*(log *n*), if *m* = *poly*(*n*), and $O\left (\frac {\log m}{\log \log m - \log \log n}\right )$, if *m* ≫ *n*. An *ex-post* BBiE protocol also exists for the Bayesian multicast game resulting in constant PoA (see Table 2 for a summary).

**Table 2:** PoA of BBiE protocols

	BBiE protocols/posted prices	
	Set cover	Multicast
Complete information	1	1
Bayesian	$O(n/\log n)$	*O*(1)

#### Posted Prices

(Section 5). For the Bayesian (and stochastic) settings, ex-post BBiE cannot be obtained by anonymous prices. We first discuss limitations of other concepts, such as BBiE with “high” probability or bounded possible excess and deficit. Then, we examine prices that result in *ex-ante* BBiE. We present anonymous prices with the same upper bounds as the BBiE protocols, for the unweighted set cover and for the multicast games, respectively. We stress that oblivious solutions may not be sufficient to guarantee low PoA for anonymous posted prices, in contrast to the BBiE protocols. This is because it is not clear anymore how to enforce players to choose desirable strategies, since *anonymous* prices are available to anyone. The reason that they exist here is due to the specific properties of the oblivious solution.

Regarding the weighted set cover game, we are only able to provide *semi*-anonymous prices with the same bounds; by semi-anonymous we mean that the prices for each player do not depend on her identity, but only on her type. We leave the case of anonymous prices as an open question. We remark that in all cases, posted prices induce *dominant strategies* for the players. Finally, for the poly-time determinable prices, we give tight lower bounds.

#### Prior-Independent Mechanisms

(Section 6). Clearly, the above BBiE protocols and posted prices depend on the prior distribution. Prior-independent mechanisms are also of high interest and in Section 6 we discuss their limitations.

#### Complete Information Setting

(Section 7). We further study the complete information setting (see Tables 1 and 2). By using either BBiE protocols or *anonymous* posted prices, we enforce the strategy profile of the optimum solution. Note that, while trying to bound the PoA, computational issues are not of primary concern. However, if we stick to protocols that can be determined in polynomial time, we can upper bound the PoA of set cover and multicast games by *O*(log *k*) and 1.39, respectively, where *k* is the number of players. Moreover, we argue that there are *no* anonymous prices, computed in polynomial time, for the set cover game, with PoA= *o*(log *k*).

### Related Work

There is a vast amount of research in cost-sharing games and so, we only mention some of the most related. Moulin and Shenker [57] studied cost-sharing games under mechanism design context; they characterized the budget-balanced and group strategyproof mechanisms and identify the one with minimum welfare loss. In similar context, other papers considered (group)strategy proof and efficient mechanisms and relaxed the budget-balanced constraint; Devanur, Mihail and Vazirani [34] and Immorlica, Mahdian and Mirrokni [50] studied the set cover game under this context showing positive and negative bounds on the fraction of the cost that is covered.

Regarding network design games, there is a long line of works mainly focusing on fair cost allocation (Shapley cost-sharing mechanism), originated by [5]. Anshelevich et al. [5] showed a tight Θ(log *k*) bound on the PoS for directed networks, while for undirected networks several variants have been studied [11, 12, 22, 24, 35] but the exact value of PoS still remains an open problem. For multicast games, Li [56] proved an upper bound of *O*(log *k*/loglog *k*), while for broadcast games, Fiat et al. [39] proved an *O*(loglog *k*) upper bound which was improved to constant due to Bilò, Flammini and Moscardelli [13]. The PoA of some special equilibria has been also studied in [17, 21].

Chen, Roughgarden and Valiant [23] were the first to study the design aspects for this game, identifying the best protocol with respect to the PoA and PoS in various cases, followed by [62] for parallel links, [40, 43, 54] for weighted congestion games, [29, 47, 59] for network games, [41] for routing games and [52] for resource allocation. The Bayesian Price of anarchy was first studied in auctions by [26]; see also [58] for routing games, and [61] for the PoS of Shapley protocol in cost-sharing games.

Close in spirit to our work is the notion of Coordination Mechanisms [25] which provide a way to improve the PoA in cases of incomplete information. Similar to our context, the designer has to decide in advance game-specific policies, without knowing the exact input. Such mechanisms have been used for scheduling problems under the objective of minimising the makespan [2, 7, 16, 49, 53] or minimising the sum of players’ costs [1, 9, 33], as well as for simple routing games [10, 28].

Posted prices have been used for pricing in large markets. Kelso and Crawford [4] and Gul and Stacchetti [46] proved the existence of prices, for gross substitute valuations, that clear the market efficiently. Pricing bundles for combinatorial Walrasian equilibria was introduced by Feldman, Gravin and Lucier [37], who showed that half of the social welfare can be achieved. In a follow-up work [38], they considered Bayesian combinatorial auctions and they could guarantee half of the optimum welfare, by using anonymous posted prices. Dynamic pricing schemes has been used by Cohen, Eden, Fiat and Jez [31] in several online settings to induce the same performance as the best online algorithm, and by Cohen-Addad, Eden, Feldman and Fiat [32] in matching markets in order to achieve the optimal social welfare, for any tie breaking rule. For maximizing the revenue with posted price mechanisms see [3, 6, 8, 14, 18*–*20].

We further discuss some related work to the underlying problems that we consider here, the set cover and the minimum Steiner tree problems. Both problems are very well studied and known to be in NP-complete. The best known approximations are *O*(log(*k*)) [30] (by using a simple greedy algorithm) and 1.39 [15]; in fact, for the set cover problem, Feige [36] showed that no improvement by a constant factor is likely. Research has been done regarding the stochastic model, Grandoni et al. [45] showed a roughly *O*(log *nm*) tight bound for the set cover problem and Garg et al. [42] gave bounds on the approximation of the stochastic online Steiner tree problem. A slightly different distribution is the independent activations. Shmoys and Talwar [60] demonstrated randomized and deterministic algorithms with constant approximations for the universal TSP problem, and Christodoulou and Sgouritsa [29] studied the multicast game presenting an ordered protocol with constant PoA.

## Model

### Cost-Sharing Protocol

In the cost-sharing games, we consider that there are *k* players who are interested in a set of resources, *R* = {*r*_1_, …, *r*_*m*_}. Each resource *r* carries a cost *c*_*r*_. Whenever a subset of players uses a resource *r*, they are charged some cost-share, defined by a cost-sharing (resource-specific) method *ξ*. A cost-sharing protocol Ξ decides a cost-sharing method for each resource. In accordance with previous works, [23, 29, 62], the following are some natural properties that Ξ needs to satisfy:

- *Stability*: The induced game has always a *pure* (Bayes) Nash equilibrium.
- *Separability*: The cost shares of each resource *r* are completely determined by the set of players that choose it.
- *BBiE*: In any pure (Bayes) Nash equilibrium profile, the cost shares of the players choosing *r* should cover exactly the cost of *r*.

For the rest of the paper, by *k* we denote the number of players and by *n* the number of different types of the players, i.e. in the set cover game, |*U*| = *n*, and in the multicast game, |*V* | = *n*.

### Information Models

We study several information models, from the point of view of the designer and of other players, regarding the knowledge of players’ type. A player’s type is some resource: in the set cover game, it is some element from *U* that needs to be covered, and in the multicast game, it is some vertex of *G*, on which the player’s terminal lies, and requires connectivity with the root *t*. The parameters of the game is known to both the protocol designer and the participants. To be more specific, the tuple $(U,\mathscr {F},c)$ in the set cover game and the underlying (weighted) graph in the multicast game are commonly known.

The information models that we consider are the following:

- *Complete Information*: The types of the players are common knowledge, i.e. they are known to all players and to the designer.
- *Stochastic/A priori*: The players’ types are drawn from some product distribution *D* defined over the type set (*U* for set cover and *V* for multicast). The actual types are unknown to the designer, who is only aware of *D*. However, the players decide their strategies by knowing other players’ types.
- *Bayesian*: The players’ types are drawn from some product distribution *D* defined over the type sets. Both the designer and the players know only *D*. The players now decide their strategies by knowing only *D* and not the actual types. A natural assumption is that every player knows her own type.

We assume that the players’ types are distributed i.i.d. (*D* = *π*^*k*^) and the type of each player is drawn independently from some probability distribution *π* : *R* → [0, 1], with ${\sum }_{r\in R} \pi (r) = 1$; *R* is either *U* in the set cover game or *V* in the multicast game. For simplicity we write *π*_*r*_ instead of *π*(*r*).

### Price of Anarchy (PoA)

Let *opt*(**t**) be the optimum solution given the players’ types **t**, and *NE*(**t**) and *BNE* be the set of pure Nash equilibria and pure Bayesian Nash equilibria, respectively. We denote the cost of any solution/strategy profile **s** as *c*(**s**). Then, the *Price of Anarchy* (PoA) for the complete information, stochastic and Bayesian settings is defined, respectively, as:

$$\begin{array}{@{}rcl@{}} PoA &=& \underset{\mathbf{s} \in NE(\mathbf{t})}{\underset{\mathbf{t}}{\max}}\frac{c(\mathbf{s})}{c(opt(\mathbf{t}))}; \qquad PoA = \max\limits_{D}\frac{\mathbb{E}_{\mathbf{t}\sim D}[\max_{\mathbf{s} \in NE(\mathbf{t})}c(\mathbf{s})]}{\mathbb{E}_{\mathbf{t}\sim D}[c(opt(\mathbf{t}))]};\\ PoA &=& \underset{D, \mathbf{s} \in BNE}{\max}\frac{\mathbb{E}_{{\mathbf{t}\sim D}}[c(\mathbf{s}(\mathbf{t}))]}{\mathbb{E}_{\mathbf{t}\sim D}[c(opt(\mathbf{t}))]}. \end{array} $$

## Lower Bounds for Budget-Balanced Protocols

In this section, we show the lower bounds of budget-balanced protocols, for the Bayesian setting.

### **Theorem 1**

*The Bayesian or stochastic PoA of any budget-balancedprotocol, for the unweighted set cover game, is* Ω(*n*)*.*

### *Proof*

Consider n players and n elements/types *U* = (1, …, *n*) and the family of sets $\mathscr {F}=\{F_{1}=\{1\}, F_{2}=\{2\},\ldots F_{n}=\{n\},F_{all}=U\}$ with unit costs. Suppose that *π* is the uniform distribution over *U*. Then the probability that element *i* is drawn as the type of at least one player is

$$q_{i} = 1-\left( 1-\frac 1n\right)^{n} \geq 1-\frac 1e. $$

By using any budget-balanced protocol, it is a (Bayes) Nash equilibrium if each player of type *i* chose set *F*_*i*_. Her cost-share does not exceed 1, while by deviating to *F*_*all*_ her cost-share becomes 1. The expected cost of that equilibrium is *nq*_*i*_ = Ω(*n*), whereas the optimum solution (all players choose the set *F*_*all*_) has cost 1. □

### **Theorem 2**

*The Bayesian PoA of any budget-balanced protocol, for the multicast game,* *is* ${\Omega }(\sqrt {n})$ *.*

### *Proof*

**Fig. 1:**
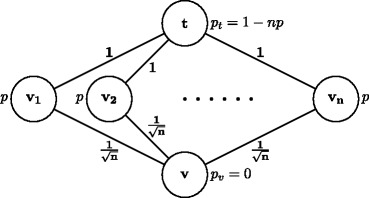
Lower bound on the PoA of any budget-balanced protocol

Consider the graph of Fig. 1. We set $p = 1-\left (1-\frac 1{\sqrt {n}} \right )^{\frac 1n}$, such that the probability that vertex *v*_*i*_ is drawn as the type of at least one player is $q_{i} = 1-\left (1-p\right )^{n} = \frac 1{\sqrt {n}}$. We claim that, for any budget-balanced protocol, it is a Bayes-Nash equilibrium if any player with type *v*_*i*_ uses the direct edges (*v*_*i*_, *t*).

Indeed, if player *i* uses any other path (*v*_*i*_, *v*, *v*_*j*_, *t*) her cost-share will be at least $\frac 2{\sqrt {n}}+(1-q_{j})= 1+\frac 1{\sqrt {n}}$, which is greater than her current cost-share of at most 1. The expected social cost and optimum are respectively

$$\mathbb{E}[SC]=\sum\limits_{i} q_{i} = \sqrt{n} ; \qquad \mathbb{E}[Opt] \leq \sum\limits_{i} q_{i}\cdot \frac 1{\sqrt{n}} + 1 = n\frac 1n + 1 = 2. $$

So, the Bayes PoA is at least $\frac 12 \sqrt {n}$. □

## BBiE Protocols

In this section we drop the requirement of budget balance and instead we consider a more general class of cost-sharing protocols $\mathscr {C}$, where the requirement is to preserve the budget balance in the equilibrium. For the rest of the paper, by *h* we denote a very high value with respect to the parameters of the game. *h* should be larger than the total cost-share of any player by using any budget-balanced protocol. It is sufficient that $h > \max _{j} c_{F_{j}}$ for the set cover game and $h > {\sum }_{e\in E}c_{e}$ for the multicast game. To show our results we will use known oblivious algorithms of the corresponding optimization problems and we will enforce their solution by applying appropriate cost-sharing protocols (or posted prices in Section 5); e.g. choices, not consistent with this solution, are highly expensive.

In an optimization problem, an oblivious algorithm assigns an action for each input component, based on the prior distribution, and *independently of the realization* of all other input components. Take as an example the multicast game, where the actions of an input (source) corresponds to the paths connecting the source to the root. An oblivious solution, maps each vertex to some path that connects it to the root, and is used in *any realization* of the input that contains this source. We associate the types of the players to the input components of the problem, and then we would like to enforce the players to follow the action decided by the oblivious algorithm for their type.

### **Theorem 3**

*LetG be any cost-sharing game and* π *the underlying optimization resource allocationproblem. Given any oblivious algorithm of* π *with approximation ratioρ,there exists a cost-sharing protocol*${\Xi } \in \mathscr {C}$*forG with PoA*= *O*(*ρ*)*.*

### *Proof*

Suppose that *R*_*i*_ is the set of the resources allocated by the oblivious algorithm to the input component that serves as the type of some player *i*. Even though it is not quite correct, we will say that *R*_*i*_ are the resources allocated to player *i*. Let *S*_*r*_ be the set of players to whom resource *r* is allocated.

Then Ξ assigns the following cost-share to any player *i* for choosing any resource *r*, when the set of players choosing *r* is *S*,

$$\xi_{r}(i,S) = \left\{ \begin{array}{l l} c_{r}/|S| & \text{if } i \in S_{r} \\ h & \text{if } i \notin S_{r} \\ 0 & \text{otherwise} \end{array} \right. $$

Note that Ξ assigns equal shares restricted to *S*_*r*_ and a high value *h* for other players. In fact, instead of equal shares we could use any budget-balanced protocol restricted to *S*_*r*_, for instance any generalized weighted Shapley protocol (for definition see [44]).

Note that any player *i* using a resource *r* ∉ *R*_*i*_ should pay *h*. By the definition of *h*, this is *strictly* more than ${\sum }_{r^{\prime }\in R_{i}} c_{r^{\prime }}$, which is the maximum she may pay if she deviates to *R*_*i*_. Therefore, the only Nash equilibria are for each player *i* to choose some subset of *R*_*i*_. This results to a PoA which is at most the same with the approximation ratio of the optimization problem, so PoA = *O*(*ρ*). Moreover, by the construction of Ξ, BBiE holds. □

The following corollaries hold for both the Bayesian and the stochastic setting.

### Set Cover Game

Grandoni et al. [45] studied the stochastic problem, and they showed two mapping algorithms for the oblivious set cover problem (one for the *unweighted* problem which is *length-oblivious* and one for the *weighted* problem which is *length-aware*), which are almost *O*(log *mn*)-competitive.

For completeness, we give the two algorithms of [45] in Algorithms 1 and 2. These algorithm are applied in the case of uniform distribution and then a reduction applies to generalize the results. 
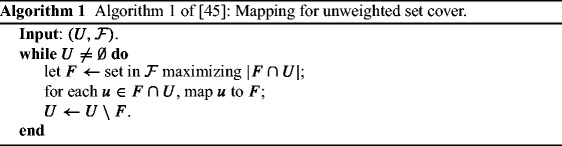

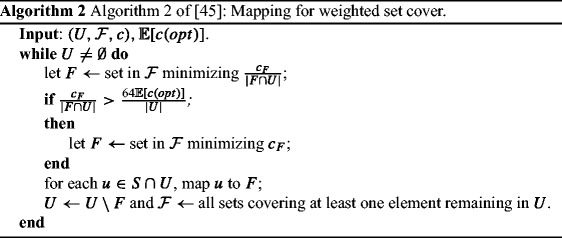


Theorem 3 implies the following corollary by using the results of [45].

### **Corollary 1**

*In the unweighted and weighted set cover game, there existlength-oblivious protocol*${\Xi }_{1} \in \mathscr {C}$*andlength-aware protocol*${\Xi }_{2} \in \mathscr {C}$*,respectively, both computed in polynomial time, and with PoA ofO*(log *n*)*,ifm* = *poly*(*n*)*,and*$O\left (\frac {\log m}{\log \log m - \log \log n}\right )$*,ifm* ≫ *n.*

### Multicast Game

Garg et al. [42] showed a constant approximation on the online Steiner tree problem. The idea is the following: sample a set *S* from the distribution *π*^*k*^ over the vertices and construct a minimum Steiner tree (or a constant approximation). Then connect each other vertex with its nearest vertex from *S* via shortest path. That way we end up with a spanning tree *T* (standard derandomization techniques can apply [29, 60, 63]). *T* defines a single path from each vertex to the root and this is an oblivious strategy for each players’ type. By using Theorem 3 and any constant approximation of the minimum Steiner tree (the best known is by [15]), the following corollary holds.

### **Corollary 2**

*In the multicast game, there exists*${\Xi }\in \mathscr {C}$*with PoA*= *O*(1)*.*

## Posted Prices

In this section, we show how to set *anonymous* or *semi-anonymous* prices for the resources. Ex-post BBiE cannot be obtained by using anonymous posted prices, as the following example illustrates. Instead, we require *ex-ante* BBiE. Furthermore, the same example serves to demonstrate that other natural variations of budget balance cannot be very promising: a) BBiE with “high” probability, b) bounded possible excess and deficit. Example 1 indicates that *any* anonymous posted prices may result in BBiE with probability at most $O(1/\sqrt {k})$ and that *no* posted prices can guarantee good bounds on possible excess and deficit, i.e. for *any* posted prices, there are cases where the total shares for some resource are either at least $\sqrt {k}$ or at most $1/\sqrt {k}$ of the resource’s cost.

### *Example 1*

Consider k i.i.d. players whose type is the uniform distribution over two elements *e*_1_, *e*_2_ in set cover or two vertices *v*_1_, *v*_2_ in multicast. In the set cover game, there are only two subset of unit cost, $\mathscr {F} = \{\{e_{1}\},\{e_{2}\}\}$. In the multicast game there are only two edges, (*v*_1_, *t*) and (*v*_2_, *t*), of unit cost. The question that arises in both cases is how to set a price on a resource *r* of unit cost, when each player may use it with probability 1/2. Let q be the price for resource *r*. If 1/*q* is not an integer in {1, …, *k*}, then budget balance appears with zero probability. So, suppose that 1/*q* = *k*^′^∈{1, …, *k*}, then budget balance appears only when *k*^′^ players use resource *r* and this happens with probability, $\text {Pr}[\# \; players = k^{\prime }] = \binom kk^{\prime } \left (\frac 12\right )^{k^{\prime }} \left (1-\frac 12\right )^{k-k^{\prime }} \leq \binom k {\lfloor k/2 \rfloor } 1/2^{k} < 1/{\sqrt {k}}. $ Furthermore, for any price q for resource *r*, if $q \geq 1/\sqrt {k}$ then, in the case that all players use *r*, the total shares sum up to at least $k\cdot 1/\sqrt {k} = \sqrt {k}$. On the other hand, if $q < 1/\sqrt {k}$ then, in the case that only one player uses *r*, her share is at most $1/\sqrt {k}$. This means that we cannot guarantee good bounds on any possible excess and deficit.

For the rest of the section we define *k*_*A*_ to be the expected number of players having type in *A* and ${k_{A}^{1}}$ to be the expected number of players having type in *A*, given there exists at least one such player:

1 $$\begin{array}{@{}rcl@{}} k_{A} &=& \mathbb{E}_{\mathbf{t}}[|i:t_{i} \in A|] = k\sum\limits_{i\in A} \pi_{i} ; \\ {k_{A}^{1}} &=& \mathbb{E}_{\mathbf{t}}[|i:t_{i} \in A| \text{ given } |i:t_{i} \in A| \geq 1] =\frac{k{\sum}_{i\in A} \pi_{i}}{1-\left( 1-{\sum}_{i\in A} \pi_{i}\right)^{k}} . \quad \end{array} $$

### Set Cover Game

To determine anonymous prices for the unweighted set cover game, we first state Lemma 1 to be used in stability arguments.

### **Lemma 1**

*For anya* > *b* > 0 *and integerk* ≥ 2*,*$\frac {a}{1-(1-a)^{k}} > \frac {b}{1-(1-b)^{k}}$*.*

### *Proof*

We prove the lemma by mathematical induction on *k*. For *k* = 2,

$$\frac{a}{1-(1-a)^{2}} = \frac a{2a-a^{2}} = \frac 1{2-a} > \frac 1{2-b} = \frac{b}{1-(1-b)^{2}}. $$

Suppose that the statement holds for *k* − 1, i.e. $\frac {a}{1-(1-a)^{k-1}} > \frac {b}{1-(1-b)^{k-1}}$. We show the equivalent inequality $\frac {1-(1-a)^{k}}a < \frac {1-(1-b)^{k}}b$,

$$\begin{array}{@{}rcl@{}} &&\frac {1-(1-a)^{k}}a = \frac {1-(1-a)^{k-1}(1-a)}a = \frac {1-(1-a)^{k-1} +a(1-a)^{k-1}}a \\ \!&=&\! \frac {1-(1-a)^{k-1}}a +(1-a)^{k-1} < \frac {1-(1-b)^{k-1}}b +(1-b)^{k-1} = \frac {1-(1-b)^{k}}b . \end{array} $$

□

### **Theorem 4**

*In the unweighted set cover game, there exist length-oblivious and anonymous prices (computed in polynomial time) with PoAO*(log *n*)*,ifm* = *poly*(*n*)*,and*$O\left (\frac {\log m}{\log \log m - \log \log n}\right )$*,ifm* ≫ *n.*

### *Proof*

In order to set the prices, we run the greedy algorithm of [30] and at each step we set the price for the selected set. Algorithm 3 describes this procedure. 
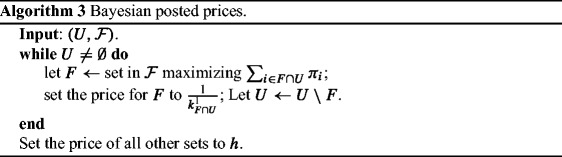


We first argue that there exists a *unique* Bayes-Nash equilibrium, where each player *i* chooses the set picked earlier by Algorithm 3 and covers her. For that it is sufficient to show that for any two sets *A* and *B*, such that ${\sum }_{i\in A} \pi _{i} > {\sum }_{i\in B} \pi _{i}$, ${k_{A}^{1}} > {k_{B}^{1}}$; so, if some player is covered by both *A* and *B*, the price set for *A* should be less than the price set for *B* and the player prefers *A* that is picked by Algorithm 3 before *B*.

By using (1), we need to show that $ \frac {k{\sum }_{i\in A} \pi _{i}}{1-\left (1-{\sum }_{i\in A} \pi _{i}\right )^{k}} > \frac {k{\sum }_{i\in B} \pi _{i}}{1-\left (1-{\sum }_{i\in B} \pi _{i}\right )^{k}},$ which is true for *k* ≥ 2, due to Lemma 1 by setting $a={\sum }_{i\in A} \pi _{i}$ and $b={\sum }_{i\in B} \pi _{i}$. Note that for *k* = 1, there exists only one player in the game and this is a trivial case to solve.

Next notice that, given that a set *F* is chosen by some player, the expected number of players paying for it is ${k_{F}^{1}}$, resulting in ex-ante BBiE. As for the PoA, Grandoni et al. [45] analyzed the performance of Algorithm 3, for the stochastic problem. They didn’t consider any prices, instead they mapped each player to the first set considered by the algorithm and they used the mapping in order to form a set cover. Their cover though coincides with the equilibrium solution and therefore their results immediately provide bounds on the PoA. □

### **Theorem 5**

*In theweighted set cover game, there existlength-aware andsemi-anonymous prices (computed in polynomial time) with PoAO*(log *n*)*,ifm* = *poly*(*n*)*,and*$O\left (\frac {\log nm}{\log \log m - \log \log n}\right )$*,ifm* ≫ *n.*

### *Proof*

By using the mapping of Algorithm 2 (Algorithm 2 of [45]), let *S*(*F*) be the set of elements mapped to set F. For each set $F \in \mathscr {F}$, set the price to be $\frac {c_{F}}{k_{S(F)}^{1}}$, for any player whose type is in *S*(*F*), and let the price be *h* for any other player. Such way, in the Bayes-Nash equilibrium, each player chooses the set, to which she is mapped by Algorithm 2. Grandoni et al. [45] showed that the expected cost of that mapping is *O*(log *n*), if *m* = *poly*(*n*), and $O\left (\frac {\log m}{\log \log m - \log \log n}\right )$, if *m* ≫ *n*, away from the expected cost of the optimum solution. Those also serve as upper bounds on the PoA of the induced game. Finally note that those prices satisfy ex-ante BBiE, for the same reasons as the unweighted case. □

We complement our results by providing tight lower bounds for poly-time determinable prices.

### **Proposition 1**

*Form* = *poly*(*n*)*,there maynot exist anonymous posted prices for theunweighted set cover, or semi-anonymous posted pricesfor the weighted set cover, computed in poly-time, withPoA*= *o*(log *n*)*,unlessNP* ⊆ *DTIME*(*n*^*O*(log log *n*)^)*.*

### *Proof*

For any set cover problem ${\Pi } = (U,\mathscr {F}, c)$, we consider the game G with |*U*| players where each one is associated with a different element. Consider the stochastic or Bayesian game, where *k* ≫ *n*, and k is sufficiently large such that the probability that each element is the type of some player converges to 1. Then, we apply the prices on the stochastic G. It is easy to see that we can compute a Nash equilibrium in polynomial time, *O*(*nm*); players choose, among the sets that covers them, some with minimum price. All the chosen sets define a set cover for *U*. If there exist posted prices computed in polynomial time with PoA= *o*(log *nm*) = *o*(log *n*), this would imply a polynomial time algorithm for the set-cover problem with approximation ratio *o*(log *n*). However, by [36], no polynomial time algorithm for the set cover problem can approximate the optimal solution by *o*(log *k*), unless *NP* ⊆ *DTIME*(*n*^*O*(log log *n*)^), which results in a contradiction. □

### **Proposition 2**

*Form* ≫ *n,there maynot exist anonymous prices for unweighted setcover, or semi-anonymous prices for weighted set cover, withPoA*$=o\left (\frac {\log m}{\log \log m - \log \log n}\right )$*.*

### *Proof*

On the contrary, suppose that such prices exist. Then, they would determine a mapping from the elements to the sets, meaning each element is covered by a specific set. The expected cost of the sets that cover the sampled elements would be $o\left (\frac {\log m}{\log \log m - \log \log n}\right )$ away from the expected cost of the optimum solution. This contradicts the lower bound given by [45] (Theorem 4.2). □

### Multicast Game

We construct a spanning tree *T* in the same way as in Section 4 and we use it to set the posted prices (computed in polynomial time).

### **Theorem 6**

*In the multicast game, there exist anonymous posted prices withPoA*= *O*(1)*.*

### *Proof*

For each edge *e* ∈ *E*(*T*), let *V* (*e*) be the set of vertices that are disconnected from the root *t* in *T* ∖{*e*}. We set the price for each *e* ∈ *E*(*T*) as $c_{e}/k_{V(e)}^{1}$. For each *e* ∉ *E*(*T*), the price is set to *h*. In the equilibrium each player chooses the path that connects her terminal with *t* via *T*. The constant PoA follows by [42] and the approximation of [15]. The expected total prices for *e* ∈ *E*(*T*) is $k_{V(e)}^{1} c_{e}/k_{V(e)}^{1} = c_{e}$, if e is used, and 0 otherwise, resulting in ex-ante BBiE. □

## Prior-Independent Mechanisms

The design of prior-independent mechanisms is a more difficult task, as the objective now is to identify a single mechanism that always has good performance, under any distributional assumption. In this section, we show limitations of prior-independent mechanisms even for the restricted class of i.i.d. prior distributions.

### BBiE Protocols

Satisfying BBiE with prior-independent protocols highly restricts the class of cost-sharing protocols and seems hard for natural classes of distribution, e.g. i.i.d., to find ex-post BBiE protocols with low PoA. Regarding the weighted set cover game with i.i.d. distributions, we can construct a lower bound of ${\Omega }(\sqrt {n})$ for all prior-independent mechanisms, which are ex-post BBiE.

### **Theorem 7**

*In the* *weighted set cover game, any prior-independent, ex-post BBiE* *protocol* ${\Xi } \in \mathscr {C}$ *has PoA* $={\Omega }(\sqrt {n})$ *.*

### *Proof*

Consider n players, *n* + 1 elements/types *U* = {0, 1, …, *n*} and the family of sets $\mathscr {F}=\{F_{0}, F_{1}, {\ldots } F_{n}, F_{all}\}$, with *F*_*j*_ = {*j*}, $c_{F_{j}}= 1$ for all *j*, and *F*_*all*_ = {1, …, *n*}, $c_{F_{all}}=\sqrt {n}$. Note that 0 is covered only by *F*_0_, serving as a dummy set.

Given a BBiE, prior-independent protocol Ξ, suppose that there exists some *F*_*j*_, *j* ≠ 0, where Ξ is not budget-balanced, i.e. there exists a set of players *S*, such that if only *S* chooses *F*_*j*_, the sum of their cost-shares are different from 1. Consider the prior distribution *D*_1_ = *π*^*n*^ with *π*(0) = *π*(*j*) = 1/2 and *π*(*j*^′^) = 0 for any *j*^′^∉{0, *j*}. With positive probability, 1/2^*n*^, all player of *S* have type *j* and all other players have type 0. If all players of *S* choose *F*_*j*_ in any pure Bayes-Nash equilibrium, ex-post BBiE is violated. So, there exists at least one player from *S* such that, whenever her type is *j*, she chooses *F*_*all*_ (and this happens with probability 1/2) which results in PoA$={\Omega }(\sqrt {n})$.

Suppose now that Ξ is budget-balanced for any *F*_*j*_, where *j* ≠ 0. Let I be the set of players such that whenever *i* ∈ *I* is the only player choosing *F*_*all*_, Ξ doesn’t charge $\sqrt {n}$ to *i*. Consider the prior distribution *D*_2_ = *π*^*n*^ with *π*(0) = 1/2 and *π*(*j*) = 1/2*n* for all other *j*. With positive probability, 1/(2^*n*^*n*), player i’s type is some *j* ≠ 0 and all other players’ type is 0. If for any type *j* ≠ 0 player i chooses *F*_*all*_ in any Bayes-Nash equilibrium, ex-post BBiE is violated. Therefore, for any player *i* ∈ *I*, whenever her type is *j*, she chooses *F*_*j*_.

We claim that the strategy profile, where any player *i* with type *t*_*i*_ chooses $F_{t_{i}}$ is a Bayes-Nash equilibrium. For any player *i* ∈ *I* there is no other valid strategy. For each player *i* ∉ *I*, whenever *t*_*i*_ ≠ 0, player i always pays at most 1 (due to budget balance in $F_{t_{i}}$), whereas if she deviates to *F*_*all*_ she pays $\sqrt {n}$.

Each element *j* ≠ 0 is a type of at least one player with probability $1-\left (1-\frac 1{2n}\right )^{n}\geq 1-\frac 2e$, giving an expected cost of Ω(*n*) in the equilibrium. The expected optimum is at most $1+\sqrt {n}$ by using only *F*_0_ and *F*_*all*_ and so PoA$={\Omega }(\sqrt {n})$. □

### Posted Prices

Setting prior-independent posted prices cannot guarantee any BBiE, even ex-ante. Consider the set cover game (similar example exists for the multicast game) with *n* players, *n* elements and two subsets of unit costs, one containing element 1 and the other containing the rest. Suppose now that we post a price *q* for the first subset. If $q \leq 1/\sqrt {n}$, for the uniform prior distribution, the expected number of players with type 1, given that there exists at least one, is $\frac {n\cdot 1/n}{1-(1-1/n)^{n}} \leq \frac {e}{e-1}$. The expected cost shares for the first set are $O (1/\sqrt {n})$, meaning that its cost is undercovered by a factor of ${\Omega }(\sqrt {n})$. If $q > 1/\sqrt {n}$, consider the prior *D* = *π*^*n*^, where *π*(1) = 1 and *π*(*j*) = 0 for all *j* ≠ 1. All players choose the first set and their total shares are $n \cdot 1/\sqrt {n} = \sqrt {n}$ which exceeds the set’s cost by a factor of $\sqrt {n}$. So, there is no way to avoid an over/under-charge of a resource by a factor better than ${\Theta }(\sqrt {n})$.

## Complete Information

In the complete information setting, the input now is known and therefore for any feasible solution we can consider oblivious strategies. If $\mathscr {F}^{\prime }\subseteq \mathscr {F}$ is any feasible solution with cost $c(\mathscr {F}^{\prime })$ and $\mathscr {F}^{*}\subseteq \mathscr {F}$ is the optimum solution with cost $c(\mathscr {F}^{*})$, then by Theorem 3 we can enforce the solution $\mathscr {F}^{\prime }$ and get a PoA of $c(\mathscr {F}^{\prime })/c(\mathscr {F}^{*})$. In the following we consider $\mathscr {F}^{\prime }$ as either the optimum solution or an approximation. Note that, while trying to bound the PoA, computational issues are not of primary concern.

### Set Cover Game

By considering $\mathscr {F}^{\prime }$, as either the optimum solution or its *O*(log *k*)-approximation of the greedy algorithm, where *k* is the number of players to be covered, and by using Theorem 3 we get the following corollary.

### **Corollary 3**

*For the (weighted) set cover game there exists a protocol*${\Xi } \in \mathscr {C}$*,that can be defined inexponential time, with PoA*= 1 *and a protocol*${\Xi } \in \mathscr {C}$*,that can be defined inpolynomial time, with PoA*= *O*(log *k*)*.*

Next we show that there exist posted prices that can be computed in polynomial time with PoA = *O*(log *k*). We show that, under the restriction of setting the prices in polynomial time, this bound is tight. Then we drop the constrain of defining the prices in polynomial time, and we define posted prices with PoA = 1.

### **Theorem 8**

*There exist posted prices satisfying BBiE, that can be set inpolynomial time, for the (weighted) set cover game, with PoA*= *O*(log *k*)*.*

### *Proof*

Let *k*_*e*_ be the number of players with type *e* ∈ *U* and *X* ⊆ *U* be the set of elements that needs to be covered, i.e. *k*_*e*_ = 0 if and only if *e* ∉ *X*. Clearly ${\sum }_{e\in X} k_{e}$ equals *k*, the total number of players. To set the prices we run Algorithm 4. 
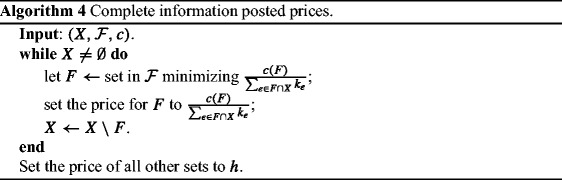


There exists a unique Nash equilibrium, where each player *i* chooses the set picked earlier by Algorithm 4 and covers her, let it be *F*^*i*^. This is because any other set *F* that covers *i* and picked after *F*^*i*^ should have at least the same value $\frac {c(F)}{{\sum }_{e \in F\cap X} k_{e}}$ with $\frac {c(F^{i})}{{\sum }_{e \in F^{i}\cap X} k_{e}}$ at the time that *F*^*i*^ was picked. After processing *F*^*i*^, *F* ∩ *X* is diminished by at least 1, and so the price of *F* should be strictly higher that the price of *F*^*i*^.

In order to show the bound on the PoA, we consider a reduction from the set cover game $G=(U, X,\mathscr {F},c,\mathbf {t})$, where **t** are players’ types, to a set cover problem ${\Pi } = (U^{\prime },\mathscr {F}^{\prime },c^{\prime })$, such that the PoA equals the approximation ratio of π. *U*^′^ is derived by *X*, after replacing each of its elements, *e*, by *k*_*e*_ copies $e_{1}, \ldots , e_{k_{e}}$. For each set $F \in \mathscr {F}$, we construct a set $F^{\prime } \in \mathscr {F}^{\prime }$ of the same cost $(c_{F}=c^{\prime }_{F^{\prime }})$, first by erasing all elements belonging to *U* ∖ *X*, and then by replacing each of its remaining elements, *e*, by *k*_*e*_ copies $e_{1}, \ldots , e_{k_{e}}$. We assume that each player *i* ∈{1, …, *k*_*e*_} of type *e* is associated with element *e*_*i*_ of the constructed set cover problem.

It is easy to see that the greedy algorithm on π chooses the sets in the same order with Algorithm 4. Therefore, the approximation ratio for π equals the PoA of *G*. Notice that |*U*^′^| = *k* and since the approximation ratio of the greedy is *O*(log *k*), the bound on the PoA follows. Note further that the sum of the prices for each such set used in the Nash equilibrium equals the cost of the set, that results in BBiE as desired. □

### **Proposition 3**

*There maynot exist posted prices, that are computedin polynomial time, for the set cover game, with PoA*= *o*(log *k*)*,unlessNP* ⊆ *DTIME*(*n*^*O*(log log *n*)^)*.*

### *Proof*

On the contrary, suppose there exist posted prices computed in polynomial time with PoA= *o*(log *k*). This would impply a polynomial time algorithm for the set cover problem with approximation ratio of *o*(log *k*), but this is a contradiction due to [36]. □

### **Theorem 9**

*There exist posted prices satisfying BBiE, computed inexponential time, for the (weighted) set-cover game, with PoA*= 1*.*

### *Proof*

As in the proof of Theorem 8, let *k*_*e*_ be the number of players with type *e* ∈ *U* and *X* ⊆ *U* be the set of elements/players that need to be covered. Moreover, let $\mathscr {F}^{*}\subseteq \mathscr {F}$ be the optimum solution, found in exponential time. To set the prices we run Algorithm 4 but for input $(X,\mathscr {F}^{*})$. We set the prices for the rest of the sets $\mathscr {F}\setminus \mathscr {F}^{*}$ equal to *h*.

By using similar arguments as in the proof of Theorem 8, in the (unique) Nash equilibrium, each player chooses the set picked earlier by Algorithm 4 and covers her. The prices for each set used in the Nash equilibrium equal the cost of the set resulting in BBiE. The difference here is that the Nash equilibrium uses only the sets of $\mathscr {F}^{*}$, resulting in PoA = 1. □

### Multicast Game

Similarly with the set cover game, we can easily get the following corollary by using Theorem 3, for the multicast game. For the second part, we use the 1.39-approximation algorithm of [15].

### **Corollary 4**

*For the multicast game there exists a cost-sharing protocol*${\Xi } \in \mathscr {C}$*,that can be defined inexponential time, with PoA*= 1 *and a cost-sharing protocol*${\Xi } \in \mathscr {C}$*,that can be defined inpolynomial time, with PoA*≤ 1.39*.*

We next use posted prices and show that the PoA is constant for the case of multicast game. By using the 1.39-approximation algorithm of [15], the PoA is constant even if we require the prices to be computed in polynomial time.

### **Theorem 10**

*For the multicast game, there exist postedprices, computed in polynomial time, with PoA*≤ 1.39 *and posted prices, computed in exponential time, with PoA*= 1*.*

### *Proof*

Let *S* ⊆ *V* be the set of players’ terminal and *T* be the solution that approximates the minimum Steiner tree on the requested vertices *S* ∪{*t*} derived by the 1.39-approximation algorithm of [15]. If we drop the requirement of computing the prices in polynomial time, *T* is the minimum Steiner tree. For each edge *e* ∈ *E*(*T*), let *k*_*e*_ be the number of players that are disconnected from *t* in *T* ∖{*e*}. We set the price for *e* ∈ *E*(*T*) as *c*_*e*_/*k*_*e*_. For each *e* ∉ *E*(*T*), we set the price to be *h*. In the Nash equilibrium each player will choose the unique path that connects her source with *t* in *T*, since any other path has high cost of at least *h*. Obviously, the players cover exactly the cost of each used edge and the cost of the Nash equilibrium equals the cost of *T*. □

## Appendix: A Set Cover Reduction

The following multicast (directed) network cost-sharing game models the set cover game. Consider a directed bipartite graph with *U* and $\mathscr {F}$ as the two sets of vertices. For each pair of vertices *i* ∈ *U* and $F_{j} \in \mathscr {F}$, we add a directed edge (*i*, *F*_*j*_) if and only if *i* ∈ *F*_*j*_; the cost of such an edge is set to 0. Each vertex *i* of *U* is associated with the terminal *t*_*i*_ of some player. We further add an extra vertex *t* as the common destination and we add a directed edge (*F*_*j*_, *t*) for every $F_{j} \in \mathscr {F}$, with cost $c_{F_{j}}$.

All strategies are two length paths and for each player/element *i* ∈ *U* the space of their strategies are all the paths where their middle vertex is a set that *i* belongs to. The cost-sharing protocol for each (*F*_*j*_, *t*) edge determines exactly the cost-shares for the players that choose *F*_*j*_. From the lower bound of Chen et al. [23] (Proposition 4.12), the following corollary can be trivially derived.

### **Corollary 5**

*The PoA of the (unweighted) set cover cost-sharing game for the complete information**setting is* Ω(*n*)*.*

## Footnotes

1 In ex-post budget balance we require budget balance in every realization of the game. If the *expected* excess and deficit are zero, the budget balance is called ex-ante.
